# *Er81 *is a downstream target of Pax6 in cortical progenitors

**DOI:** 10.1186/1471-213X-8-23

**Published:** 2008-02-28

**Authors:** Tran Cong Tuoc, Anastassia Stoykova

**Affiliations:** 1 Max-Planck-Institute for Biophysical Chemistry, Göttingen, Germany; 2 DFG, Center of Molecular Physiology of the Brain (CMPB), Götingen, Germany

## Abstract

**Background:**

Although the transcription factor Pax6 plays an essential role in neurogenesis, layer formation and arealization in the developing mammalian cortex, the mechanisms by which it accomplishes these regulatory functions are largely unknown. *Pax6 *and the ETS family transcription factor *Er81*, which is presumed to play a role in the specification of a sublineage of layer 5 projection neurons, are expressed with a prominent rostrolateral-high to caudomedial-low gradient in cortical progenitors. In the absence of functional *Pax6*, progenitors do not express *Er81 *and the rostrolateral cortex lacks *Er81*-positive layer 5 neurons. In this study, we investigated the transcriptional regulation of *Er81 *and provide evidence that *Er81 *is a direct target of Pax6.

**Results:**

We identified and analyzed the regulatory function of an evolutionarily conserved upstream DNA sequence in the putative mouse *Er81 *promoter. Three potential Pax6 binding sites were identified in this region. We found that the presence of one of these sites is necessary and sufficient for full activation of the *Er81 *promoter in Pax6-transfected HeLa cells, while other still unknown factors appear to contribute to *Er81 *promoter activity in cortical progenitors and neuronal cells. The results suggest that endogenous Pax6, which is expressed at the highest level in progenitors of the rostrolateral cortex, exerts region-specific control of *Er81 *activity, thus specifying a subpopulation of layer 5 projection neurons.

**Conclusion:**

We conclude that the genetic interplay between the transcription factors, *Pax6 *and *Er81*, is responsible, in part, for the regional specification of a distinct sublineage of layer 5 projection neurons.

## Background

In the mammalian neocortex (pallium), neurons with striking morphological and functional diversity are organized radially in six layers, and tangentially into numerous functional domains. Only recently have the molecular and cellular mechanisms that guide the process of corticogenesis responsible for this organization begun to be resolved [1,2]. The main source of cortical projection neurons is the population of pluripotent radial glial progenitors (RG), which divide asymmetrically at the apical surface of the ventricular zone (VZ) and generate both neuronal and glial progeny [3]. After midgestation, RG generate neuronal progenitors, termed intermediate or basal progenitors (BPs), that divide symmetrically at the basal surface of the VZ and in the subventricular zone (SVZ). Thus, while the asymmetric division of RG progenitors gives rise to progeny with distinct cell fates, the symmetric division of BPs primarily modulates the number of cells in previously established neuronal cell lineages [4]. The projection neurons of the lower (6 and 5) and upper (4–2) layers are generated predominantly from early (E12-E14) or late (E15-E18) progenitors in the two germinative zones, VZ and SVZ, respectively. Although generated during a specific developmental window, each neuronal layer consists of molecularly distinct neuronal subtypes that arise sequentially [5-7]. For instance, the majority of layer 5 neurons that extend corticospinal projections express the transcription factor, *Er81 *[8], while another set of layer 5 neurons, marked by the expression of the homeodomain transcription factor, *Otx1*, make connections with the superior colliculus and pons [5]. Thus, laminar fate is presumably determined not only by the timing of neuronal origin during distinct developmental stages, a process controlled by environmental cues [9], but is also critically dependent on intrinsic mechanisms that control the molecular phenotype of the neuronal sublineages [5]. The mechanisms that control the restricted expression of molecular determinants in distinct classes of neurons during corticogenesis remain unknown. Our previous results, as well as those of other groups, have indicated that neurogenic RG progenitors are intrinsically specified by the expression of *Pax6 *[10]. In the absence of Pax6, as exemplified by *Pax6/Small eye *homozygous *(Sey/Sey*) embryos, cortical progenitors produce less than half the normal number of neurons; conversely, retroviral-mediated Pax6 overexpression in cortical progenitors *in vitro *results in expanded production of neuronal progeny [11,12]. *Pax6 *is strongly expressed in early progenitors and, although it directly regulates the activity of the neuronal determination gene, *Ngn2 *[13]. *Ngn2- *and *Pax6-*controlled genetic programs appear to specifically and separately determine the neuronal fate of lower and upper neurons [14]. Thus, *Ngn2 *knock out (KO) and *Sey/Sey *embryos exhibit selective misspecification of lower and upper cortical layer neurons, respectively [14]. Intriguingly, however, in the absence of Pax6, cortical progenitors fail to express the layer 5-specific marker, *Er81*, which instead exhibits enhanced ectopic expression in the SVZ [15]. We have noted qualitatively similar mispatterning of *Er81 *expression in the rostrolateral cortex in the cortex-specific conditional *Pax6 KO *mice at juvenile and adult stages (T.C.T., A.S. unpublished observations), prompting us to examine possible genetic interactions between the two transcription factors, Pax6 and Er81.

Here we report the identification of a 2-kb promoter sequence of the mouse *Er81 *gene that drives *Er81 *expression in a subpopulation of cortical layer 5 neurons. We demonstrate that Pax6 directly controls *Er81 *activity in both cortical progenitors and in a subset of layer 5 projection neurons. The results further suggest that the cell fate specification of *Er81*-positive layer 5 neurons in the rostrolateral cortex is a Pax6-dependent process.

## Results

### Pax6 binds to the putative *Er81 *promoter

**Figure 1:**
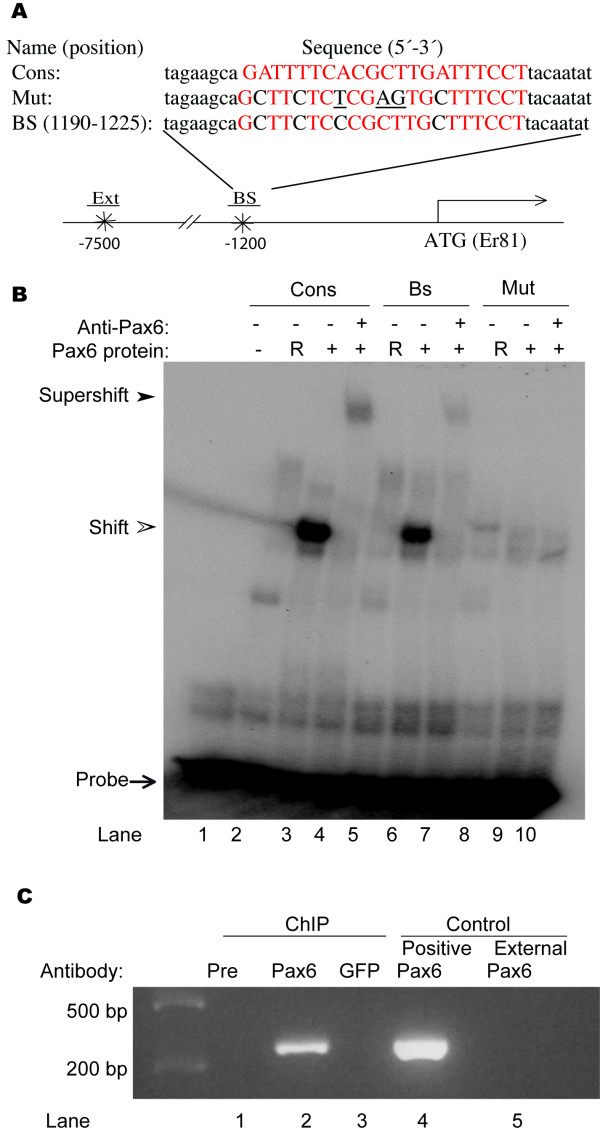
**Pax6 bind specifically to a putative *Er81 *promoter**. (A) The schematic depicts the putative *Er81 *promoter, showing the relative positions of a potential Pax6-binding site (BS) and an external control sequence used for ChIP assays (see below). A comparison of the DNA sequences for the potential Pax6-binding site identified in the putative *Er81 *promoter (at position -1190-1225), the perfect Pax6-binding site (Cons), and the mutated site (Mut) is also indicated. (B) EMSA analysis of Pax6 binding to ^32^P-labeled probes corresponding to the potential Pax6 binding site, a perfect Pax6-binding site, and a mutated site. The arrows, open arrowhead and closed arrowhead indicate free probes, probe-protein complexes, and probe-protein-antibody complexes, respectively. Binding of Pax6 to its perfect Pax6-binding site (lane 3) and potential binding site in the putative *Er81 *promoter (lane 6) is clearly detectable. Probe binding is not observed using the TNT rat reticulocyte lysate (RRL) as a protein control (lanes 2, 5). Binding specificity was confirmed by pre-incubating with an anti-Pax6 antibody, which resulted in a super-shifted DNA-Pax6 protein-Pax6 antibody complex (lanes 4, 7). Binding of Pax6 was completely abolished by mutation of the Pax6 binding-site sequence in the *Er81 *promoter (lanes 9, 10). (C) ChIP assay. Pax6 antibodies precipitated chromatin containing the Pax6 binding site in the region -1322 to -1040 of the *Er81 *promoter (lane 2), but were unable to precipitate chromatin isolated from a region outside of the Pax6 binding site (lane 4). Preimmune serum (lane1, Pre) or GFP (lane3) antibodies failed to precipitate chromatin.

DNA sequences with significant gene regulatory functions are highly conserved during evolution. A comparison of the *Er81 *locus from mouse, rat, chimpanzee and human revealed the existence of a highly conserved sequence of approximate 2 kb in the 5' region of the putative *Er81 *promoter. To identify potential Pax6 consensus binding sites [16] in this region, we utilized the sequence analysis package GCG [17]. We discovered three potential Pax6 binding sites located at positions -113 to 148, -1190 to 1225, and -1530 to 1565 upstream of the mouse *Er81 *gene, each of which contained three, four or five mis-matches relative to the consensus Pax6 binding sequence [16]. Using an electrophoretic mobility shift assay (EMSA) and *in vitro*-translated Pax6 protein, we examined Pax6 binding to these three potential binding sites. The results indicate that Pax6 bound with low affinity to the Pax6 binding site at position -1190 to -1225, but not to any of the other putative sites (Fig. 1B, lanes 3 and 6 and data not shown). To determine whether this low-affinity binding was specific, we pre-incubated binding mixtures with an anti-Pax6 antibody and found that protein-bound, radiolabeled probes were supershifted, confirming the presence of a DNA-Pax6 protein-Pax6 antibody complex (Fig. 1B lane 7). Pax6 binding was also completely abolished after mutating the binding site sequence in the *Er81 *promoter (Fig. 1A,B lanes 9, 10). Together, these findings indicate that Pax6 interacts specifically with the -1190-1225 regulatory sequence of the *Er81 *promoter.

To determine whether this binding site is occupied by Pax6 protein *in vivo*, we performed chromatin immunoprecipitation (ChIP) assays using mouse E15.5 cortical extracts. A 282-bp fragment located at -1322 to -1040 of the *Er81 *promoter encompassing the Pax6 binding site was precipitated from chromatin by the Pax6 antibody (Fig. 1C, lane 2), but not by pre-immune serum (Pre) or GFP antibody (Fig. 1C, lane 1 and 3). Furthermore, the Pax6 antibody was not able to precipitate a control chromatin fragment isolated from a region outside of the putative *Er81 *promoter (Fig. 1C, lane 5). These data indicate that Pax6 protein binds specifically to the putative *Er81 *promoter both *in vitro *and *in vivo*.

Significantly, the identified Pax6 binding site is also present in the 2-kb upstream-region of the *Er81 *gene of mouse, rat, chimpanzee and human (data not shown) and in *zebrafish *[18], suggesting that this binding site is evolutionally conserved.

### Cell type-dependent regulation of *Er81 *promoter activity

**Figure 2:**
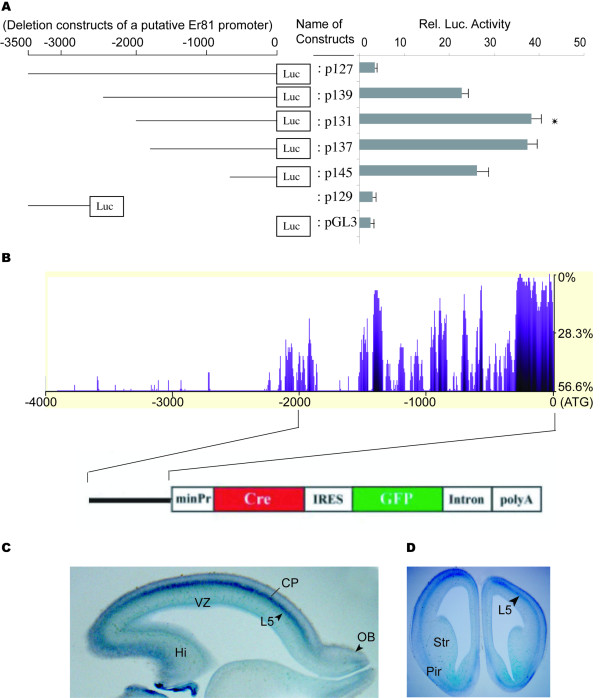
**Characterization of the *Er81 *promoter**. (A) Diagram to the left indicates the relative size of deletion constructs used in the reporter assay. The horizontal lines represent deleted fragments of the putative *Er81 *promoter. Diagram to the right shows the corresponding reporter activities of the indicated deletion constructs in primary cortical cell cultures, expressed as mean ± standard deviation (error bars) in each assay. The asterisk masks the construct, p131 with the highest luciferase activity among other deletion constructs. (B) Multiple sequence alignment of the mouse, rat, chimpanzee, and human *Er81 *genes was performed using the ClustalW algorithm implemented in the eShadow web application [55]. The x- and y-axes indicate the distance (in base pairs) to the starting codon (0) and percentage variation, respectively. The resulting alignment indicates a highly conserved sequence of approximate 2 kb upstream of the *Er81 *gene for all species examined. Schematic representation of the vector used to generate a Cre-transgenic mouse line in which the expression of *Er81 *is driven by the identified 2-kb *Er81 *promoter sequence. The *Er81 *promoter was subcloned upstream of the β-globin minimal promoter (minPr) in a plasmid containing a DNA fragment encoding Cre [53]. This construct allows the *Er81 *promoter to simultaneously drive expression of Cre via the β-globin minimal promoter, and a GFP reporter sequence via an *IRES *sequence. (C) After crossing with Gtrosa26^tm1Sho ^reporter mice [27], LacZ staining of E16.5 forebrains isolated from double-transgenic *Er81Cre *Gtrosa26 mice showed recombination in the L5, and the VZ of cortex, striatum, piriform cortex, and olfactory bulb (C, cross section; D, sagittal section). CP, Cortical plate; Hi, Hippocampus; L5, Layer 5; OB, Olfactory bulb; Pir, Piriform cortex; Str, Striatum; VZ, Ventricular zone.

We next used deletion analysis to determine the minimal sequence requirements for *Er81 *promoter activity. A 3.5-kb fragment from the 5' region of the mouse *Er81 *gene and a series of deletion fragments were subcloned into luciferase reporter plasmids and transfected into mouse primary embryonic (E12.5) cortical cell cultures, as described below (Fig. 2A). The *Er81(wt)-Luc *construct (p131), containing a 2-kb region of genomic DNA upstream of the *Er81 *translation initiation codon, was most active in subsequent luciferase assays.

**Figure 3:**
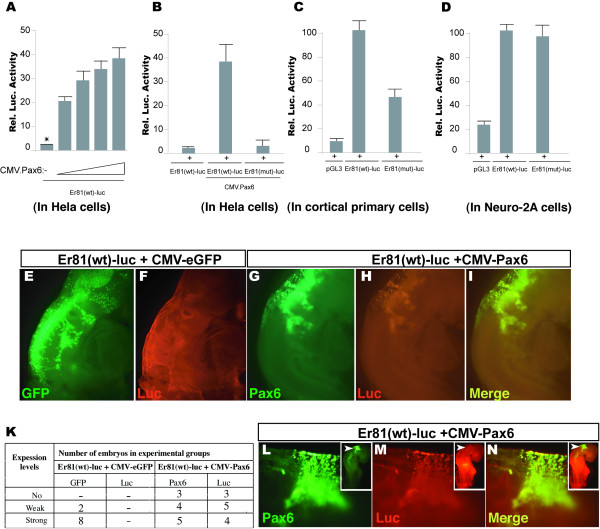
**The Pax6 binding site is required for full activity of the putative *Er81 *promoter**. (A) Exogenously expressed Pax6 activated a co-transfected *Er81 *promoter-reporter construct in *HeLa *cells in a dosage-dependent manner. (B) Mutation of the Pax6 binding site completely abolished Pax6-dependent luciferase reporter activity of the putative *Er81 *promoter in HeLa cells. The plasmid combinations used for transfections are indicated. (C) Mutation of the Pax6-binding site led to a significant decrease in *Er81 *promoter activity in mouse embryonic (E12.5) primary cortical cultures. (D) The putative Er81 promoter exhibited high activity in Neuro-2A cells (compare *Er81(wt)-luc *construct and control pGL3). Deletion of the Pax6 binding site did not affect activation of the putative Er81 promoter in Neuro-2A cells (compare *Er81(wt)-luc *and *Er81(mut)-luc *constructs). (E-K) Pax6 activated an *Er81 *promoter-reporter construct *in ovo*. (E) GFP immunoreactivity demonstrates the high efficiency of the electroporation method. (E/F) After co-electroporation of *Er81(wt)-Luc *and *CMV-eGFP*, no luciferase-positive cells were detected. (G-I) Co-electroporation of *Er81(wt)-Luc *and *CMV-Pax6 *promoted expression from the *Er81 *promoter-luciferase reporter construct in hindbrain of chick embryos. (K) The table shows the number of embryos used for immunohistochemical analyses and the results obtained with the indicated antibodies. The strength of the immunohistochemical signal is designated as no, weak or strong staining. Note that the images shown in (E-H) represent strong staining for GFP, Luciferase and Pax6 antibodies. (L/N) Consistently, co-electroporation of *Er81(wt)-Luc *and *CMV-Pax6 *in isthmic region, where Pax6 does not express, led to up-regulation of the *Er81 *promoter-controlled luciferase reporter. Arrows in images for the whole embryo indicated a region where plasmids were injected. Luciferase activity is expressed as mean ± standard deviation (error bars) in each assay. *Er81(wt)-Luc*, wild-type putative *Er81 *promoter-reporter construct; *Er81(mut)-Luc*, mutated putative *Er81 *promoter-reporter construct.

To assess the ability of Pax6 to activate the *Er81 *promoter, we transfected HeLa cells, which lack endogenous Pax6 [19], with *Er81(wt)-Luc *alone (control) or together with the Pax6-expression plasmid, *CMV-Pax6 *(Fig. 3A). The control cells exhibited very low basal levels of luciferase activity, whereas co-transfection of *Er81(wt)-Luc *with increasing amounts of *CMV-Pax6 *led to robust, concentration-dependent increases in luciferase activity (Fig. 3A).

To determine whether *Pax6 *is also able to activate the *Er81 *promoter *in vivo*, we performed *in ovo *co-electroporation experiments. *Er81(wt)-Luc *and *CMV-Pax6 *constructs (or a *CMV-Gfp *empty vector control), were electroporated into the hindbrain of Hamburger and Hamilton (HH) stage 11–12 chick embryos. After 2 days, embryos were examined immunohistochemically for expression of Pax6, GFP and luciferase reporter. After the forced expression of Pax6 in VZ progenitors of the hindbrain, a marked expression of the *Er81*-luciferase reporter was detected as compared to the controls (Fig. 3E/I). In developing neural tube *Er81 *is expressed endogenously only in differentiated motor and proprioceptive sensory neurons, including a subpopulation of neurons of the inferior olive in the hindbrain [20,21]. Upon electroporation, the injection in the forth ventricle, DNA (through the CMV-Pax6 construct) is incorporated in VZ progenitors and their descendents, seen as strongly luciferase-positive cells leaving the neural tube (Fig. 3H). Consistent results were also obtained when the forced expression of Pax6 was tagged predominantly into the isthmic region, normally being negative for endogenous Pax6 expression (Fig. 3L/M). Together, these results indicate that forced expression of Pax6 can trans-activate the *Er81 *promoter in both, a cell culture system and a live embryo.

To further examine the functional significance of the single Pax6 binding site identified in the *Er81 *promoter, we mutated the site in the *Er81(wt)-Luc *plasmid from the original 5' CCCGCT 3' sequence to 5' CTCGAG 3', which does not bind Pax6, generating the *Er81(mut)-Luc *plasmid (Fig. 1A,B). We then used a reporter assay to assess the Pax6-dependent transactivation of both wild type and mutated constructs. Accordingly, HeLa cells were transfected with *Er81(wt)-Luc *or *Er81(mut)-Luc *with or without cotransfection of the Pax6 expression construct, *CMV-Pax6*. As expected, the activity of the wild-type promoter construct was enhanced by Pax6 overexpression, which increased luciferase activity almost 20-fold compared to the reporter activity in control cells. Mutation of the Pax6-binding site in the putative *Er81 *promoter completely abolished Pax6-dependent activity in the reporter assay (Fig. 3B). These data suggest that, in Pax6-transfected HeLa cells, a single biologically active Pax6-binding site in the *Er81 *promoter is sufficient to support full promoter activity.

To study how the occupation of the binding site by Pax6 in the *Er81 *promoter is influenced by the presence of endogenous proteins intrinsic to progenitor cells, we evaluated *Er81 *promoter activity in primary embryonic (E12.5) cortical cultures. More that 90% of the RC2+ RG progenitors express Pax6 at this stage [10,22]. The cells were electroporated with either *Er81(wt)-Luc, Er81(mut)-Luc *or empty pGL3 plasmid (control) and cultured for 3 days *in vitro *(3DIV) in a chemically defined culture medium (see Materials and Methods; [23]). Under these conditions, progenitor proliferation and neuronal differentiation *in vitro *mimic *in vivo *neurogenesis [10,24]. Notably, in contrast to *Pax6-*transfected HeLa cells, where transfection of the *Er81(mut)-Luc *construct completely abolished *Er81 *promoter activity, approximately 42% of the residual luciferase activity remained in primary cortical cultures transfected with this mutant promoter construct (Fig. 3C). These findings indicate that the Pax6-binding site in the *Er81 *promoter is an important, but possibly not the sole, cis-acting element responsible for regulating the activity of the *Er81 *promoter in cortical primary cultures.

Pax6 is expressed in RG progenitors, but is also present in some differentiated neurons in the adult brain, including some amygdalar nuclei [25]. Upon differentiation, primary cortical cultures contain a mixture of progenitors and different cell types, including neurons and glial cells. To examine the function of the Pax6 binding site identified in the *Er81 *promoter in a homogeneous neuronal cell population, we performed *Er81 *reporter assays using the neuronal cell line, Neuro-2A, which expresses little or no Pax6 [26]. Neuro-2A cells were transfected with either *Er81(wt)-Luc, Er81(mut)-Luc *or pGL3 constructs and cultured for 2 days. In agreement with the results obtained in primary cortical cells, transfection of Neuro-2A cells with the *Er81(wt)-Luc *construct caused almost a four-fold increase in luciferase reporter activity compared to that in pGL3-transfected control cultures (Fig. 3D). Remarkably, and in contrast to the experiments with *Pax6-*transfected HeLa cells and mixed primary cortical cultures, transfection of Neuro-2A cells with *Er81(mut)-Luc *construct had no effect on *Er81 *promoter activity (Fig. 3D). As noted above, Pax6 binds with low affinity to the *Er81 *promoter, suggesting that high levels of *Pax6 *expression may be required to exert transcriptional control on *Er81*. Under the culture conditions used, *Pax6 *expression in mixed primary cortical cultures is restricted to the RG progenitors cells. Collectively, these findings strongly suggest that the Pax6 binding site identified in the *Er81 *promoter is essential for activation of *Er81 *in cortical progenitors, while regulation of *Er81 *expression in neuronal cells may depend on other factors.

### The 2-kb *Er81 *promoter sequence drives appropriate expression of *Er81 *in the cortex of *Er81Cre *transgenic mice

To obtain definitive evidence that the promoter sequences identified in the *Er81 *gene is capable of correctly driving the endogenous expression of *Er81 *in the developing brain, we developed an *Er81Cre *transgenic mouse. This line expresses a DNA construct containing a 2.0-kb fragment of the *Er81 *promoter placed downstream of a human β-globin minimal promoter, followed by a Cre recombinase DNA sequence, and IRES and GFP reporter sequences (Fig. 2B). *Er81Cre *transgenic mice were crossed with mice from a reporter line, Gtrosa26^tm1Sho ^[27], which contain a loxP-flanked DNA sequence of "stopper" fragment positioned upstream of the β-galactosidase-neomycin phosphotransferase fusion gene (β-geo). β-geo is expressed only after Cre-mediated excision of loxP-flanked DNA sequences, and thus double-reporter transgenic Gtrosa26^tm1Sho^/Cre lines provide a region-specific report of the occurrence of Cre excision by the specific Cre line used [27]. The analysis of LacZ activity at E16.5 in different *Er81Cre;Gtrosa26*^tm1Sho ^double-transgenic founders revealed expression of the reporter in the VZ and layer 5 of the cortex, striatum, piriform cortex and olfactory bulb, a result that is in agreement with the known pattern of *Er81 *expression in the mouse telencephalon (Fig. 2C,D; [18,28]). We therefore conclude that the 2-kb *Er81 *promoter region is necessary and sufficient for *Er81 *expression in the mouse telencephalon.

## Discussion

Accumulating evidence suggests that the process of cortical layering, during which progenitor cells in the VZ and SVZ generate neurons destined to specific cortical layers, depends on both the temporal sequence of progenitor cell origination during a specific developmental stage and the expression of layer-determinant genes. In addition, neurons of a specific layer exhibit diversity in number, morphology and axonal connectivity across different functional domains of the cortex. Thus, the two fundamental processes of corticogenesis, layering and arealization, seem to be closely related. According to the current view, the functional regionalization of the cortex involves intrinsic mechanisms, controlled by the combinatorial expression of transcription factor gradients in the progenitors, and extrinsic cues provided by the ingrowing thalamocortical axons at late developmental stages and after birth [2,29].

In the developing cortex, *Pax6 *is expressed in RG progenitors in a rostrolateral-high to caudomedial-low gradient [30,31] and plays essential roles in cortical neurogenesis, and arealization and layer formation [10,12,14,25,30,32-37]. Accordingly, abolishing Pax6 function leads to defects in cortical molecular regionalization as observed in the *Pax6/Small eye *brain, where the rostral cortical area shrinks and caudal areas expand [38,39]. Furthermore, there is a decrease in the number of neurons in the *Pax6*-defficient cortex, in which the neuronal subsets of the upper cortical layers appear to be specifically missing [14,34]. In the current study, we provide the first evidence that Pax6 may determine the neuronal identity of subsets of layer 5 projection neurons by controlling the expression of *Er81 *in pallial progenitors.

*Er81 *is a member of the Pea3 subfamily of the ETS transcription factor family [40] that is expressed in cortical progenitors at the mid- to late stages of neocortical development in mouse [28], rat and monkey [41]. In the mouse cortex, *Er81 *transcripts are first detected at E13 in the VZ of the rostrolateral pallium; expression is maintained in a subset of the pyramidal cells in the lower part of layer 5 in later embryonic stages and in the mature brain. [41]. ETS proteins have been shown to contribute to the specification of various cell types in vertebrates and invertebrates [42]. The documented role of *Er81 *in the specification of dendritic arborization of proprioceptive sensory neurons in the spinal cord [43] suggests the possibility that *Er81 *might be involved in the neuronal subtype specification of projection neurons in the cortex [18,28]. Similar to *Pax6, Er81 *is also expressed in a prominent, graded manner in pallial progenitors, reaching its highest levels in the rostrolateral cortex [28,44,45]. In the absence of Pax6, *Er81 *expression in the VZ of the rostrolateral cortex (where endogenous Pax6 expression is highest) is essentially undetectable, both in *Sey/Sey *mutant [15] and in the juvenile cortex of the conditional cortex-specific *Pax6KO *mutant, (T.C.T and AS, unpublished data). We show here that Pax6 binds with low affinity to a single binding site in the *Er81 *promoter, and further show that Pax6 protein produces dose-dependent increases in the activity of this promoter. Collectively, these findings suggest that *Pax6 *controls the generation of *Er81+ *layer 5 neurons in a dose- and region-specific manner, predominantly in the motor and frontoparietal cortex. Remarkably, similar region- and dose-dependent regulation of *Er81 *expression by the neurogenic factor, *Ngn2*, was demonstrated in *Ngn2-KO *mice, in which *Er81 *expression is specifically affected only in the rostral cortex [14]. *Ngn2 *is a direct Pax6 downstream target gene whose expression is regulated by high levels of Pax6 expression only in progenitors of the rostral pallium [13,35]. Additional experiments will be required to determine whether *Er81 *expression in the rostrolateral cortex is regulated coordinately by *Pax6 *and *Ngn2 *or, alternatively, whether *Er81 *acts as a downstream regulator in the *Pax6-Ngn2 *pathway.

We found that the Pax6-binding site identified in the 2-kb promoter fragment of *Er81 *was necessary for full activation of the *Er81 *promoter by Pax6 in Pax6-transfected-HeLa cells. However, mutation of the Pax6-binding site only partially reduced *Er81 *promoter activity in primary cortical cell cultures and had no effect in neurons of the Neuro-2A cell line. Given that at 3DIV, cortical primary cultures consist of approximately 45 % progenitors and 55% differentiated neurons [10,22], these findings suggest that activation of *Er81 *depends primarily on Pax6 transcriptional control in cortical progenitors, but activation/maintenance of *Er81 *expression in differentiated neurons possibly involves other regulatory factors. To identify such potential factors, we performed an *in silico *search for potential upstream binding sites and identified multiple binding sites for the transcription factors, REST/NRSF and Brn2, both of which are important in cortical neurogenesis [46,47]. These transcription factors could be important for activation and/or maintenance of *Er81 *expression in cortical progenitors and subsets of differentiated neurons.

It is interesting to note that both *Pax6*- and *Ngn2*-dependent expression of *Er81 *in RG progenitors *in vivo *is confined to progenitors of the rostrolateral cortex. To confirm the functional significance of the Pax6-dependent control sequence identified in the *Er81 *promoter, we developed a transgenic mouse line in which the 2 kb *Er81 *promoter region was placed upstream of a Cre recombinase sequence. A detailed analysis of the resulting *Er81Cre *line is a subject of a separate study. Results obtained thus far indicate, however, that the expression of the LacZ reporter in *Er81Cre:Gtrosa26*^*tm*1*Sho *^double-transgenic mice faithfully reproduces the known expression pattern of *Er81 *in the developing telencephalon, including VZ progenitors and subpopulations of L5 neurons, striatum, piriform cortex and olfactory bulb (Fig. 2C,D; also [18,28]. Furthermore, the reporter LacZ staining was much fainter in the medial than in the dorsal or lateral pallium. During the process of submitting this paper, Langevin et al [18] reported that a 2-kb region upstream of the zebrafish *Er81 *is active in the lateral, but not in the medial cortex of the mouse, and identified Pax6-binding sites in a 1.3-kb upstream region [18]. Thus, it is possible that *Er81 *promoter activity in progenitors of the dorsal and lateral pallium (rostrally) as well as in the whole caudal pallium depends on combinatorial control by Pax6 and other transcription factors. Such simultaneous binding of Pax6 and the transcription factors, Sox2 and Maf, in the δ-crystallin and glucagon promoters, respectively, significantly increases Pax6 transactivation ability [48,49]. Together, these findings strongly suggest that an evolutionarily conserved genetic interplay between Pax6 and Er81 is involved in the regional specification of progenitor identity in the developing cortex.

## Conclusion

In this paper we have shown that a direct genetic interaction between the transcription factors, Pax6 and Er81, in cortical VZ may be involved in the regional specification of neuronal subtype identity of a set of layer 5 projection neurons. The low-affinity binding of Pax6 to the 2-kb *Er81 *promoter suggests that only the high endogenous levels of Pax6 in progenitors of the rostrolateral cortex are capable of regulating *Er81 *promoter activity, an interpretation that is consistent with the reported regional inhibition of *Er81 *expression in Pax6-defficient mutants. The expression of *Er81 *in cortical germinative neuroepithelium in other regions and in mature neurons may involve regulation by other molecular determinants acting independently of, or together with, Pax6.

## Methods

### Plasmids and antibodies

CMV-Pax6 [46] was used for Pax6 expression in mammalian cells. pGL3 basic served as the backbone for luciferase reporter constructs. *Er81(mut)-Luc *was generated by site-direct mutagenesis (Quick Change, Stratagene) using *Er81(wt)-Luc *as a template.

The anti-Pax6 monoclonal (1:500, DSHB), anti-Pax6 polyclonal (1:500, BABCO), anti-GFP (1:500, Chemicon), anti-firefly Luciferase (1:100, Abcam), Alexa 488 goat anti-mouse, and Alexa 594 goat anti-rabbit (1:400, Invitrogen) antibodies were used.

### Identification of transcription factor binding sites in the *Er81 *promoter

To identify potential Pax6 binding sites within a 2-kb region of the mouse *Er81 *promoter, we utilized the sequence analysis package, GCG [17], and the previously reported Pax6 consensus-binding site [16]. MatInspector software was used to search for additional transcription factor binding sites in *Er81 *promoter [50].

### Determination of Pax6 DNA binding (EMSA)

EMSA was performed as described by Bäumer et al [51] with some modifications. Briefly, Pax6 proteins were expressed using the TNT *in vitro *transcription and translation system (Promega), according to the manufacturer's instructions. Double-stranded oligonucleotides (Fig. 1A) were end-labeled using polynucleotide kinase and gramma-^P32^ATP. The binding reaction was performed for 1 hr on ice in binding buffer (25 mM HEPES pH7.4, 10% glycerol, 75 mM NaCl, 0.25 mM EDTA, 1 mM DTT, 0.1% Nonidet P-40, 1 mM MgCl_2_, protease inhibitor cocktail) containing 0.5 μg poly-dI-dC, double-stranded oligonucleotides (with radial activity at 35000 cpm) and 10 μl of *in vitro*-translated Pax6 protein. For antibody supershift analyses, 0.5 μl of Pax6 polyclonal rabbit antibody (Babco) was added and samples were incubated for an additional 15 min. Samples were load onto 4% TAE polyacrylamide gels and electrophoresed at 10 V/cm to resolve complexes. Gels were dried and processed for autoradiography.

### Chromatin immunoprecipitation (ChIP) assay

Chromatin was extracted from E15.5 mouse cortices. ChIP assays were performed according to the kit manufacture's instructions (Upstate Biotechnology) using polyclonal Pax6 antibodies (BABCO) to immunoprecipitate Pax6-binding chromatin fragments, with pre-immune serum and polyclonal GFP antibodies (Abcam) as immunoprecipitation controls (10 μg antibody per immunoprecipitation).

### Generation of an *Er81Cre *transgenic mouse line

A 2-kb region upstream from the starting codon of the mouse *Er81 *gene was amplified as an NheI/Not fragment using the Expand Long Template PCR kit (Roche) and cloned into a Cre-IRES-GFP-Intron-pA plasmid [52,53] in pSL1180 (Pharmacia). *Er81Cre *mice were generated by pronuclear microinjection. Transgenic mice were identified by PCR analysis or GFP-fluorescence and maintained in a C57BL6/J background. Animals have been handled with permission of the Bezirksregierung Braunschweig in accordance with the German Animal Protection Law.

### Cell culture and cell transfection

HeLa cells were maintained and cultured in DMEM medium containing 10% fetal calf serum (FCS). Cells were transfected using Lipofectamine 2000 (Invitrogen), according to the supplier's instructions.

### Primary cortical cell culture and electroporation

The two telencephalic hemispheres were isolated under sterile conditions in Ca^2+^/Mg^2+^-free Hanks Balanced Salt Solution containing 10 mM HEPES (HBSS/HEPES). After washing twice with fresh HBSS/HEPES solution, cells were incubated for 18 min at 37°C in 0.25% Trypsin/EDTA (Sigma). The tissue was then dissociated mechanically by triturating with a fire-polished, serum-coated Pasteur pipette, then centrifuged for 5 min at 1000 rpm, washed and resuspended in DMEM medium plus 10% FCS. Primary cortical cells were electroporated using a nucleotransfection device (Amaxa) and then cultured for 3 days on coverslips coated with poly-D-lysine and Sato medium (DMEM, 100 μg/ml albumin, 100 μg/ml apo-transferrin, 16 μg/ml putrescine, 0.06 ng/ml progesterone, 40 ng/ml selenium, 5 μg/ml insulin, 1 mM sodium pyruvate and 2 mM L-glutamine) [23].

### Luciferase assay

HeLa cells or primary cortical cells were lysed and assayed for Luciferase activity according to the assay manufacturer's instructions (Promega).

### *In ovo *chick electroporation

Chick embryos at stage 11–12 (36–48 hr old) were electroporated as described in Marquardt et al [54]. Briefly, 2.5 μg/μl of *Er81-Luc *and 2.5 μg/μl of *CMV-Pax6 *(or *CMV-Gfp*) were co-electroporated into the hindbrain or midbrain of chick embryos. After 1–2 days, embryos were collected and analyzed using immunohistochemistry. At least 10 electroporated embryos were analyzed in each experiment.

### X-Gal staining

Embryos from timed mating were fixed and stained overnight with X-Gal at 37°C as described previously [27,53].

## Authors' contributions

AS and TCT conceived and designed the study. TCT carried out experiments. AS and TCT wrote the manuscript. All authors approved the manuscript

## References

[B1] Guillemot F, Molnar Z, Tarabykin V, Stoykova A (2006). Molecular mechanisms of cortical differentiation. Eur J Neurosci.

[B2] Mallamaci A, Stoykova A (2006). Gene networks controlling early cerebral cortex arealization. Eur J Neurosci.

[B3] Gotz M, Huttner WB (2005). The cell biology of neurogenesis. Nat Rev Mol Cell Biol.

[B4] Kriegstein A, Noctor S, Martinez-Cerdeno V (2006). Patterns of neural stem and progenitor cell division may underlie evolutionary cortical expansion. Nat Rev Neurosci.

[B5] Hevner RF, Daza RA, Rubenstein JL, Stunnenberg H, Olavarria JF, Englund C (2003). Beyond laminar fate: toward a molecular classification of cortical projection/pyramidal neurons. Dev Neurosci.

[B6] Arlotta P, Molyneaux BJ, Chen J, Inoue J, Kominami R, Macklis JD (2005). Neuronal subtype-specific genes that control corticospinal motor neuron development in vivo. Neuron.

[B7] Molyneaux BJ, Arlotta P, Menezes JR, Macklis JD (2007). Neuronal subtype specification in the cerebral cortex. Nat Rev Neurosci.

[B8] Watakabe A, Ichinohe N, Ohsawa S, Hashikawa T, Komatsu Y, Rockland KS, Yamamori T (2007). Comparative analysis of layer-specific genes in Mammalian neocortex. Cereb Cortex.

[B9] McConnell SK, Kaznowski CE (1991). Cell cycle dependence of laminar determination in developing neocortex. Science.

[B10] Gotz M, Stoykova A, Gruss P (1998). Pax6 controls radial glia differentiation in the cerebral cortex. Neuron.

[B11] Haubst N, Berger J, Radjendirane V, Graw J, Favor J, Saunders GF, Stoykova A, Gotz M (2004). Molecular dissection of Pax6 function: the specific roles of the paired domain and homeodomain in brain development. Development.

[B12] Heins N, Malatesta P, Cecconi F, Nakafuku M, Tucker KL, Hack MA, Chapouton P, Barde YA, Gotz M (2002). Glial cells generate neurons: the role of the transcription factor Pax6. Nat Neurosci.

[B13] Scardigli R, Baumer N, Gruss P, Guillemot F, Le Roux I (2003). Direct and concentration-dependent regulation of the proneural gene Neurogenin2 by Pax6. Development.

[B14] Schuurmans C, Armant O, Nieto M, Stenman JM, Britz O, Klenin N, Brown C, Langevin LM, Seibt J, Tang H, Cunningham JM, Dyck R, Walsh C, Campbell K, Polleux F, Guillemot F (2004). Sequential phases of cortical specification involve Neurogenin-dependent and -independent pathways. Embo J.

[B15] Stenman J, Toresson H, Campbell K (2003). Identification of two distinct progenitor populations in the lateral ganglionic eminence: implications for striatal and olfactory bulb neurogenesis. J Neurosci.

[B16] Epstein JA, Glaser T, Cai J, Jepeal L, Walton DS, Maas RL (1994). Two independent and interactive DNA-binding subdomains of the Pax6 paired domain are regulated by alternative splicing. Genes Dev.

[B17] Womble DD (2000). Web-based interfaces for the GCG sequence analysis programs. Methods Mol Biol.

[B18] Langevin LM, Mattar P, Scardigli R, Roussigne M, Logan C, Blader P, Schuurmans C (2007). Validating in utero electroporation for the rapid analysis of gene regulatory elements in the murine telencephalon. Dev Dyn.

[B19] Cartier L, Laforge T, Feki A, Arnaudeau S, Dubois-Dauphin M, Krause KH (2006). Pax6-induced alteration of cell fate: shape changes, expression of neuronal alpha tubulin, postmitotic phenotype, and cell migration. J Neurobiol.

[B20] Zhu Y, Guthrie S (2002). Expression of the ETS transcription factor ER81 in the developing chick and mouse hindbrain. Dev Dyn.

[B21] Lunn JS, Fishwick KJ, Halley PA, Storey KG (2007). A spatial and temporal map of FGF/Erk1/2 activity and response repertoires in the early chick embryo. Dev Biol.

[B22] Hartfuss E, Galli R, Heins N, Gotz M (2001). Characterization of CNS precursor subtypes and radial glia. Dev Biol.

[B23] Bottenstein JE, Sato GH (1979). Growth of a rat neuroblastoma cell line in serum-free supplemented medium. Proc Natl Acad Sci USA.

[B24] Qian X, Shen Q, Goderie SK, He W, Capela A, Davis AA, Temple S (2000). Timing of CNS cell generation: a programmed sequence of neuron and glial cell production from isolated murine cortical stem cells. Neuron.

[B25] Stoykova A, Gruss P (1994). Roles of Pax-genes in developing and adult brain as suggested by expression patterns. J Neurosci.

[B26] Pinson J, Simpson TI, Mason JO, Price DJ (2006). Positive autoregulation of the transcription factor Pax6 in response to increased levels of either of its major isoforms, Pax6 or Pax6(5a), in cultured cells. BMC Dev Biol.

[B27] Mao X, Fujiwara Y, Orkin SH (1999). Improved reporter strain for monitoring Cre recombinase-mediated DNA excisions in mice. Proc Natl Acad Sci USA.

[B28] Hasegawa H, Ashigaki S, Takamatsu M, Suzuki-Migishima R, Ohbayashi N, Itoh N, Takada S, Tanabe Y (2004). Laminar patterning in the developing neocortex by temporally coordinated fibroblast growth factor signaling. J Neurosci.

[B29] Sur M, Rubenstein JL (2005). Patterning and plasticity of the cerebral cortex. Science.

[B30] Stoykova A, Gotz M, Gruss P, Price J (1997). Pax6-dependent regulation of adhesive patterning, R-cadherin expression and boundary formation in developing forebrain. Development.

[B31] Walther C, Gruss P (1991). Pax-6, a murine paired box gene, is expressed in the developing CNS. Development.

[B32] Schmahl W, Knoedlseder M, Favor J, Davidson D (1993). Defects of neuronal migration and the pathogenesis of cortical malformations are associated with Small eye (Sey) in the mouse, a point mutation at the Pax-6-locus. Acta Neuropathol (Berl).

[B33] Caric D, Gooday D, Hill RE, McConnell SK, Price DJ (1997). Determination of the migratory capacity of embryonic cortical cells lacking the transcription factor Pax-6. Development.

[B34] Stoykova A, Fritsch R, Walther C, Gruss P (1996). Forebrain patterning defects in Small eye mutant mice. Development.

[B35] Stoykova A, Treichel D, Hallonet M, Gruss P (2000). Pax6 modulates the dorsoventral patterning of the mammalian telencephalon. J Neurosci.

[B36] Tarabykin V, Stoykova A, Usman N, Gruss P (2001). Cortical upper layer neurons derive from the subventricular zone as indicated by Svet1 gene expression. Development.

[B37] Manuel M, Georgala PA, Carr CB, Chanas S, Kleinjan DA, Martynoga B, Mason JO, Molinek M, Pinson J, Pratt T, Quinn JC, Simpson TI, Tyas DA, van Heyningen V, West JD, Price DJ (2007). Controlled overexpression of Pax6 in vivo negatively autoregulates the Pax6 locus, causing cell-autonomous defects of late cortical progenitor proliferation with little effect on cortical arealization. Development.

[B38] Bishop KM, Goudreau G, O'Leary DD (2000). Regulation of area identity in the mammalian neocortex by Emx2 and Pax6. Science.

[B39] Muzio L, DiBenedetto B, Stoykova A, Boncinelli E, Gruss P, Mallamaci A (2002). Emx2 and Pax6 control regionalization of the pre-neuronogenic cortical primordium. Cereb Cortex.

[B40] Sharrocks AD (2001). The ETS-domain transcription factor family. Nat Rev Mol Cell Biol.

[B41] Yoneshima H, Yamasaki S, Voelker CC, Molnar Z, Christophe E, Audinat E, Takemoto M, Nishiwaki M, Tsuji S, Fujita I, Yamamoto N (2006). Er81 is expressed in a subpopulation of layer 5 neurons in rodent and primate neocortices. Neuroscience.

[B42] Wasylyk B, Hagman J, Gutierrez-Hartmann A (1998). Ets transcription factors: nuclear effectors of the Ras-MAP-kinase signaling pathway. Trends Biochem Sci.

[B43] Lin JH, Saito T, Anderson DJ, Lance-Jones C, Jessell TM, Arber S (1998). Functionally related motor neuron pool and muscle sensory afferent subtypes defined by coordinate ETS gene expression. Cell.

[B44] Fukuchi-Shimogori T, Grove EA (2003). Emx2 patterns the neocortex by regulating FGF positional signaling. Nat Neurosci.

[B45] Sansom SN, Hebert JM, Thammongkol U, Smith J, Nisbet G, Surani MA, McConnell SK, Livesey FJ (2005). Genomic characterisation of a Fgf-regulated gradient-based neocortical protomap. Development.

[B46] Ballas N, Grunseich C, Lu DD, Speh JC, Mandel G (2005). REST and its corepressors mediate plasticity of neuronal gene chromatin throughout neurogenesis. Cell.

[B47] Castro DS, Skowronska-Krawczyk D, Armant O, Donaldson IJ, Parras C, Hunt C, Critchley JA, Nguyen L, Gossler A, Gottgens B, Matter JM, Guillemot F (2006). Proneural bHLH and Brn proteins coregulate a neurogenic program through cooperative binding to a conserved DNA motif. Dev Cell.

[B48] Kamachi Y, Uchikawa M, Tanouchi A, Sekido R, Kondoh H (2001). Pax6 and SOX2 form a co-DNA-binding partner complex that regulates initiation of lens development. Genes Dev.

[B49] Planque N, Leconte L, Coquelle FM, Benkhelifa S, Martin P, Felder-Schmittbuhl MP, Saule S (2001). Interaction of Maf transcription factors with Pax-6 results in synergistic activation of the glucagon promoter. J Biol Chem.

[B50] Quandt K, Frech K, Karas H, Wingender E, Werner T (1995). MatInd and MatInspector: new fast and versatile tools for detection of consensus matches in nucleotide sequence data. Nucleic Acids Res.

[B51] Baumer N, Marquardt T, Stoykova A, Spieler D, Treichel D, Ashery-Padan R, Gruss P (2003). Retinal pigmented epithelium determination requires the redundant activities of Pax2 and Pax6. Development.

[B52] Ashery-Padan R, Marquardt T, Zhou X, Gruss P (2000). Pax6 activity in the lens primordium is required for lens formation and for correct placement of a single retina in the eye. Genes Dev.

[B53] Berger J, Eckert S, Scardigli R, Guillemot F, Gruss P, Stoykova A (2004). E1-Ngn2/Cre is a new line for regional activation of Cre recombinase in the developing CNS. Genesis.

[B54] Marquardt T, Ashery-Padan R, Andrejewski N, Scardigli R, Guillemot F, Gruss P (2001). Pax6 is required for the multipotent state of retinal progenitor cells. Cell.

[B55] Ovcharenko I, Boffelli D, Loots GG (2004). eShadow: a tool for comparing closely related sequences. Genome Res.

